# Japan Collaborative Cohort Study for Evaluation of Cancer Risk Sponsored by Monbusho (JACC Study)

**DOI:** 10.2188/jea.11.144

**Published:** 2007-11-30

**Authors:** Yoshiyuki Ohno, Akiko Tamakoshi, the JACC Study Group

**Affiliations:** 1 Department of Preventive Medicine/Biostatistics and Medical Decision Making, Nagoya University Graduate School of Medicine.; 2 To whom correspondence should be addressed. e-mail: yohno@med.nagoya-u.ac.jp (Yoshiyuki Ohno).; 3 See acknowledgments for the past investigators involved in the JACC Study.

**Keywords:** Japan collaborative cohort study (JACC Study), cancer, lifestyle, serum, DNA, nested case-control study, biological markers

## Abstract

This paper primarily aimed to overview the rationale for initiating the Japan Collaborative Cohort Study for Evaluation of Cancer Risk Sponsored by Monbusho (Ministry of Education, Science, Sports and Culture of Japan) (JACC Study), by comparing socio-demographic and nutritional changes that were witnessed between 1965 and 1990 in Japan, and also to describe the study design, the follow-up conditions as of the end of 1997, and the frameworks for analyzing the data of the lung, stomach, pancreas and gallbladder/bile duct, based on the approximately 8-year follow-up data. For other major sites such as cancers of the large intestine and liver, an analysis will be started in the fiscal year of 2002. This paper secondarily aimed to be cited as the basic information on the JACC Study when several publications are to be based on.

## INITIATION AND RATIONALE OF JACC STUDY

Before around 1985 in Japan, a large-scale population-based cohort study, which aimed to evaluate cancer risk modification by lifestyle, was the Six-Prefecture Cohort Study (so-called Hirayama’s cohort study)^1^^)^ alone. This study had started in 1965 (and the follow-up was terminated in 1982), involving 122,261 men and 142,857 women aged 40 years and older at entry, residing in 29 Health Center Districts in 6 prefectures (Miyagi, Aichi, Osaka, Hyogo, Okayama and Kagoshima). The baseline data on diet and such lifestyle habits as smoking and drinking were collected by direct interview of a study subject at his/her home by public health nurses or midwives, using a simple and straightforward questionnaire that included only items easy for a study subject to answer.

Everyone had witnessed and recognized dramatic changes in lifestyle and living conditions that had occurred since around 1965 in Japan, when Hirayama’s cohort study started. This recognition certainly prompted epidemiologists in Japan to conduct other population-based cohort studies^2^^)^ for evaluating cancer risk modification by remarkably changed lifestyle. Among such cohort studies there was a large-scale population-based cohort study named as “the Japan Collaborative Cohort Study for Evaluation of Cancer Risk Sponsored by Monbusho (the JACC Study)”, which has been maintained by the Monbusho Research Committee on Evaluation of Risk Factors for Cancer by Large-scale Cohort Study (Monbusho ECC^3^^)^) since 1988.

This paper will mainly aim to describe and discuss the study design and interim condition as of the end of 1997 in the JACC Study in detail, together with the present and future frameworks for analyzing the epidemiological and biological materials collected at baseline. Before the description, however, it will be worth while looking over, in order to understand the rationale for initiating the JACC study, some demographic, social and nutritional changes that had occurred between 1965 and 1990 in Japan, which corresponded to the starting time of Hirayama’s study and the JACC Study.

Smoking rate^4^^)^ among adults, defined as those aged 20 years or over, was 82.3% and 15.7% in men and women in 1965, respectively, but 60.5% and 14.3% in 1990, correspondingly. Together with the observed decrease in male smoking rate, a notable change in smoking habit was that smoking rate had decreased in all age groups in men, but increased in the age group of 20-29 years, in particular, in women. The number of cigarettes smoked per day was 19.4 in men and 12.2 in women in 1965, but 24.7 and 18.1 in 1990, correspondingly. In 1965, cigarettes with and without filter-tip were sold half and half in proportion^4^^)^, but almost no cigarettes without filter-tip were sold in 1990, and tar yield per cigarette was remarkably reduced^5^^)^ likewise in other developed countries, though with much increased number of cigarettes smoked a day. Regular drinkers including occasional ones^6^^)^ were estimated to be 43% in 1965 and 67% in 1990 among adults. Annual amount of pure alcohol consumed by one adult was 5.8 liters in 1965 and 8.3 liters in 1990. Heavy drinkers defined as those consuming 150 ml of pure alcohol per day or more were 1,028 thousands in number in 1965 and 2,124 thousands in 1990. In Japan, beer consumption has remarkably increased year by year, but such other alcoholic beverages as sake (Japanese fermented alcoholic beverage made from rice), Schochyu (distilled alcoholic beverage made from rice, wheat, sweet potatoes and others), whiskey, and spirits have been constantly consumed for the recent three decades^6^^)^.

Demographic and social changes^7^^)^ between 1965 and 1990 were also dramatic in Japan. Total population had increased from 98 to 124 million in the 2.5 decades with almost doubled proportion of those aged 65 years old and more (from 6.3% to 12.1%). Life expectancy at birth increased by 8.2 years (from 67.74 to 75.92 years) in men and by 9.0 years (72.92 to 81.90 years) in women. Changed proportion in industrial structure was rather substantial: clearly decreased (from 24.1 to 7.1%) primary industry (agriculture, forestry, fishery and livestock farming) and apparently increased (from 43.7 to 59.4%) tertiary industry (sales, banking, real estate, transportation and communication, civil workers, services, and other industries not included in the primary and secondary industry), with almost unaltered (from 31.5 to 33.4%) proportion in the secondary industry (manufacturing, mining, construction and civil engineering). Along with the changed industrial structure and a 10-fold increase (from 273,000 to 2,774,000 Yen per capita) in national income, real income per month/household had increased by 8 times (from 65,144 to 521,757 Yen) between 1965 and 1990 with a simultaneous increase in expenditure for consumption in daily life by 6.7 times (from 49,335 to 331,595 Yen), though with decreased working hours (from 197.8 to 179.4 hours per month in men and from 181.4 to 155.3 hours in women) and working days (from 23.8 to 21.2 days per month in men and from 23.2 to 20.7 days in women). In women, mean age at first marriage was delayed (from 24.5 to 25.9 years old) with an increasing proportion of those who did not marry in life, and an approximately 3-fold increase (from 11.3 to 37.4%) in the proportion of girl students attending universities or colleges was observed among girl senior high school graduates. Sanitary and daily-life environments had also substantially improved: diffusion rate changed from 1965 to 1990 being from 69.4 to 94.7% in tap water supply, from 8.3 to 44.7% in sewerage system, from 38.2 to 98.2% in electric refrigerator, from 26.8 to 98.8% in electric clearer, from 2.0 to 63.7% in room air conditioner, and from 2.6 to 77.3% in passenger car.

Dietary and nutritional intakes also substantially changed between 1965^8^^)^ and 1990^9^^)^ in Japan. A notable increase in daily dietary consumption was observed in wheat, potatoes, oils and fats, green-yellow vegetables, fruits, fishes and shellfishes, meats, eggs, milk and dairy products: in particular, more than doubled consumption in fruits (from 58.8 to 124.8g), meats (from 29.5 to 71.2g) and milk (from 48.8 to 119.5g), and 1.2 to 1.7-fold increase in other food groups mentioned above. In contrast, rice was far less consumed (from 349.8 to 197.9g), but with unchanged consumption of pulses and seaweed. In nutrient intakes, approximately doubled intake was observed in animal fat (from 14.3 to 27.5g) and vitamin A (1,324 to 2,567IU) with a 1.5-folds increase in intake of animal protein (from 28.5 to 41.4g), vitamins B_2_ (from 0.83 to 1.33mg) and vitamin C (from 78 to 120mg).

These demographic, social and nutritional changes with apparently altered smoking and drinking behaviors in the past 2.5 decades were believed to be an important and rational background for initiating another large-scale population-based cohort study such as the JACC Study in Japan, in which associations of dramatically changed lifestyle and living conditions with cancer risk would be devotedly examined.

## STUDY DESIGN AND SUBJECTS

The two major founders of the JACC Study were Kunio Aoki, Professor of Preventive Medicine, Nagoya University School of Medicine and Haruo Sugano, Director of Cancer Institute, Tokyo (as of 1988), who financially supported to organize the Monbusho ECC^3^^, ^^10^^, ^^11^^)^.

The Monbusho ECC was organized by Prof. Aoki, after the feasibility study of the two preceding years, in 1988, involving 35 epidemiologists, including epidemiology-oriented clinicians and one statistician who were interested in initiating a multicenter-collaborative cohort study on cancer, with the two essential requests of collaborating for a minimum of 10 years and providing an appropriate successor to continue the JACC Study, should the original investigator retire or resign from his position^10^^)^. The original investigators belonged to 24 different institutions (universities, hospitals or others) and had been coordinating population screening programs for chronic diseases in 45 areas throughout Japan: 3 towns in Hokkaido district, 5 towns in Tohoku district, 8 towns and 2 villages in Kanto district, one city and one town in Chubu district, 7 towns and 2 villages in Kinki district, one city and one town in Chugoku district, 4 cities, 9 towns and one village in Kyushu district, and no area in Shikoku district.

The Monbusho ECC^3^^, ^^10^^, ^^11^^)^ enrolled about 127,500 healthy inhabitants in the areas, who attended to the screening program and responded to the questionnaire in 1988-1990, as a basic cohort population, among which 46,465 men and 64,327 women (110,792 in total) were aged 40-79 years old and were to be followed up for a minimum of 10 years. Exact response rate to the screening program was not uniformly reported from all areas to the central office of the Monbusho ECC because of methodological difficulties. But it was believed to be considerably high, since the screening program has annually been conducted for long years in each area together with enthusiastic supports for the program from the inhabitants themselves and the local community authority (among the areas where the investigators enrolled the inhabitants from their total population, the response rates were reported as high as 90%).

Table 1 shows age distribution of the cohort members aged 40-79 years at enrollment and their follow-up condition as of the end of 1997 (mean follow-up period: 8.15 years).

**Table 1.** Age distribution of cohort members at entry and deaths/move-outs as of the end of 1997.

Age at entry		40-44	45-49	50-54	55-59	60-64	65-69	70-74	75-79	Total
Men										
	Total	6,002	5,806	6,322	7,695	8,429	5,518	4,024	2,669	46,465

	Alive	5,610	5,431	5,840	6,939	7,327	4,457	2,714	1,411	39,729
	(%)	93.5	93.5	92.4	90.2	86.9	80.8	67.4	52.9	85.5
	Dead	98	155	319	584	957	951	1,221	1,187	5,472
	(%)	1.6	2.7	5.0	7.6	11.4	17.2	30.3	44.5	11.8
	Moved	294	220	163	172	145	110	89	71	1,264
	(%)	4.9	3.8	2.6	2.2	1.7	2.0	2.2	2.7	2.7

Women										
	Total	7,557	7,926	9,108	10,816	11,114	8,602	5,557	3,647	64,327

	Alive	7,159	7,563	8,669	10,229	10,341	7,693	4,514	2,528	58,696
	(%)	94.7	95.4	95.2	94.6	93.0	89.4	81.2	69.3	91.2
	Dead	59	98	165	322	517	686	830	976	3,653
	(%)	0.8	1.2	1.8	3.0	4.7	8.0	14.9	26.8	5.7
	Moved	339	265	274	265	256	223	213	143	1,978
	(%)	4.5	3.3	3.0	2.5	2.3	2.6	3.8	3.9	3.1

Cohort members who deceased have been identified with an underlying cause of death by reviewing all death certificates in each area once a year under the authoritative permission from the Director-General of the Prime Minister’s Office (Ministry of Public Management, Home Affairs, Post and Telecommunications) . This verification was worked by the investigator himself with administrative assistance from public health nurses in each area, who has well acquainted themselves with inhabitants’ vital status including out-migration and their health conditions including screening findings as well. The underlying causes of death already coded according to the International Classification of Diseases and Injuries (ICD) 9^th^ version (from the baseline to 1994) and stored in the computer database were re-coded in 1999 according to the ICD 10^th^ version (after 1995), using a specifically developed computer program^12^^)^ for converting the ICD 9^th^ code to the 10^th^ code. The move-outs were also annually verified by the investigator himself with administrative assistance from public health nurses in each area by reviewing population-register sheets of the cohort members. The verification of vital status and out-migration were believed to be substantially accurate because of firmly established population registration system in Japan, but the losts to follow-up, who were believed to be negligible in proportion, could not be methodologically identified and were regarded as alive in each area. The most likely lost to follow-up would occur when a subject moved out leaving his/her population registration in the survey area. In this case, however, when he/she deceased in the area out-migrated, his/her death certificate would be transferred to the health center in which his/her original area was located, and, therefore, his/her vital status would be readily known, though with unknown an underlying cause of death. We believed that such occasions were quite rare.

The data of deaths and move-outs in all areas were annually informed, together with incident cancer sufferers in 24 areas out of the 45 areas who were identified mostly by cancer registration system, to the central office of Monbusho ECC (Department of Preventive Medicine/Biostatistics and Medical Decision Making, Nagoya University Graduate School of Medicine), and were confidentially kept as a computer database without any information which could identify an individual.

Epidemiological data^11^^)^ were routinely collected at baseline (1988-1990) by a self-administered questionnaire with written informed consent, including such demographic information as gender, date of birth, marital status and number of children; past medical history of 7 acute infectious and 11 chronic diseases and three episodes (injuries severe enough to be hospitalized, surgical operation of the abdomen and blood transfusion); past episodes of 6 chronic diseases among family members (father, mother and siblings) with his/her own birth order; such health condition in one year prior to the entry as stool frequency, sleeping hours; liability of catching cold and having diarrhea, skin rashes, intake of vitamin supplements and other (over-counter and prescribed) drugs; exercise/sports and walking hours a day in one year prior to the entry and sports activities at junior and senior high school; health check-up including stomach cancer screening in one year prior to the entry; occupation held the longest; height; weight at entry and at age of 20 years old; usual systolic and diastolic blood pressure; residential area; type of school and age at the highest educational attainment; behavioral attitude/stress; and reproductive history (women only). Smoking habits, including passive smoking, and drinking habits of alcoholic beverages and soft drinks (coffee, black tea, Japanese and Chinese tea) were asked in detail. Dietary practices and usual frequency of food intake were also inquired in the self-administered questionnaire.

Questions on dietary practices included type of usual breakfast (Japanese or Western style, tea gruel or others), regularity of the time at taking supper, number of bowls of boiled rice a day both at entry and around 30 years old, intake frequency of miso (traditional Japanese soybean paste) soup and number of cups of miso soup a day both at entry and around 30 years old, preference for salty and fatty foods, changes in intake frequency of salty and fatty foods since around 30 years old, and dietary practice of reducing total energy and salty, sugar-rich and fat-rich foods for his/her own health maintenance. Intake frequency was asked for 32 kinds of food and cake, using 5 response categories (almost none, 1-2 times a month, 1-2 times a week, 3-4 times a week and almost everyday).

These epidemiological data were re-collected by an interim questionnaire survey in 1993-1995 among approximately 37.9% (41,965 in number) of the cohort members in order to examine possible changes in lifestyle variables.

Besides the epidemiological information above, we attempted to collect blood sample from each participant at the screening. Sera (300µl: 5 aliquots per person) of about 39,000 cohort members were successfully collected and stored at -80°C in several deep freezers, mostly at the central office of Monbusho ECC. The subjects whose sera were sampled accounted for about 35% of the total cohort members aged 40-79 years old. DNA samples of about 8,000 subjects (about 6% of the total cohort members) were collected in the limited study areas, and kept at the central DNA laboratory, the Institute of Medical Science, University of Tokyo.

Our whole study design which would singularly and collectively use epidemiological and biological materials (serum only) was approved by the Ethical Board at Nagoya University School of Medicine, where the central office of Monbusho ECC is located.

## EPIDEMIOLOGICAL FEATURES OF COHORT POPULATION IN JACC STUDY

The detailed tabulation tables based on the self-administered questionnaires were already shown in the progress report^11^^)^ published in March, 1996. In this section, several epidemiological features would be summarized by re-tabulating the baseline data cleaned as of the end of 1997.

In our cohort population aged 40-79 years old at baseline (when a self-administered questionnaire was filled in), current, ex- and non-smokers accounted for 53.0%, 26.7% and 20.4% in men, and 5.7%, 1.8% and 92.6% in women, respectively. Male ex-smokers were much higher in proportion, compared to the figure (3.8%) in Hirayama’s male cohort population^1^^)^, but with similar rate to the national figure in 1990^4^^)^. The proportions of current smokers by age were 60.1%, 54.1%, 51.6% and 40.5% in the 5^th^, 6^th^, 7^th^ and 8^th^ decade in men, respectively, and 6.9%, 5.3%, 4.8% and 5.8% in women, correspondingly. Age-specific smoking rates were similar in men, but lower than the national figures in women in 1990^4^^)^.

Current, ex- and non-drinkers were, in proportion, 74.7%, 6.6% and 18.8% in men, and 24.5%, 1.8% and 73.7% in women, respectively, in our cohort population. Age-specific proportions of current drinkers were 82.1%, 78.4%, 71.3% and 60.3% in the 5^th^, 6^th^, 7^th^ and 8^th^ decade in men, respectively. Current drinkers in our male cohort population were higher in proportion, compared to the figures in general population^9^^)^, though the definition of current drinkers was a little different from ours.

Those with no habitual exercise accounted for 68.6% in men and 76.1% in women in our cohort population: being considerably lower than the national ones^9^^)^. Those who did not take breakfast accounted for 3.4% in men and 2.5% in women: being lower than the national figures^9^^)^. As for marital status, married subjects accounted for 93.5%, widowed or divorced for 4.8% and not-married (single) for 1.7% in men with corresponding figures of 82.4%, 16.0% and 1.6% in women. These figures were well comparable to those aged 40-79 years old in general population^13^^)^.

In short, our cohort population was not established by random sampling, but appeared to be similar to Japanese general population in the light of several demographic and lifestyle features, though such similarity is not necessarily the prerequisite condition, since a cohort study itself aims, in its nature, an internal comparison between those developed and those not developed the targeted outcomes in a certain period of time.

## FOLLOW-UP CONDITIONS AS OF THE END OF 1997

The central office of Monbusho ECC examined the follow-up conditions as of the end of 1997 (mean follow-up period: 8.07 years in men and 8.21 years in women).

As shown in Table 1, among 46,465 men aged 40-79 years old at baseline (when a self-administered questionnaire was filled in), 39,729 (85.5%), 5,472 (11.8%) and 1,264 (2.7%) were identified as of the end of 1997 as the alive, deceased and move-outs from the study areas, respectively, and corresponding figures were 58,696 (91.2%), 3,653 (5.7%) and 1,978 (3.1%) among 64,327 women: 98,425 (88.8%), 9,125 (8.2%) and 3,242 (2.9%) in the total cohort population in due order. The expected numbers calculated based on the Japanese population aged 40-79 years old in 1997 (as a standard) were 6468.1 in men and 4601.9 in women for total death, with the corresponding numbers of 2516.8 and 1618.3 for cancer death: indicating a ratio (O/E) of 0.846 and 0.794 for total death and 0.852 and 0.811 for cancer death in due order. Our cohort members, therefore, appeared to be less likely to die from total causes and cancer, compared to the Japanese population aged 40-79 years old. As mentioned above, those incidentally missed to be followed were included in the alive in a negligible proportion.

Total cancer deaths accounted for 39.2% (2,145) and 35.9% (1,313) in male and female total deaths (5,472 and 3,653), with 37.9% (3,458) in total deaths (9,125) in the total cohort members, as shown in Table 2. Among male cancer deaths, cancers of the lung (including bronchus), stomach and liver were the three commonest sites, accounting for 22.7% (487 in number), 21.0% (451) and 13.5% (289), respectively. In women, the three commonest sites were cancers of the stomach, lung and large intestine, accounting for 16.8% (220 in number), 12.0% (158) and 11.7% (154), respectively. Incident cancer sufferers identified and reported from 22 areas, as of the end of 1997, were 4,321 in total. Among them, cancers of the stomach, colon, lung including the bronchus, breast, rectum and liver including intra-hepatic bile duct were the six commonest sites, accounting for 26.7% (1,153 in number), 11.4% (493), 8.1% (351), 6.6% (284), 5.5% (238) and 4.7% (203), respectively. Cancers of the prostate, thyroid, urinary bladder and pancreas accounted for 3.1% (136), 3.1% (135), 3.0% (128) and 2.6% (113), respectively, following the six sites above. Such other sites of cancer as cervix uteri, esophagus and corpus uteri were 97 (2.2%), 71 (1.6%) and 67 (1.6%) in number in due order.

**Table 2.** Age distribution of total deaths and all and site-specific cancer deaths as of the end of 1997.

Age at entry in yaeas	40-44	45-49	50-54	55-59	60-64	65-69	70-74	75-79	Total	%
Total deaths	157	253	484	906	1,474	1,637	2,051	2,163	9,125	
Cancer deaths	63	112	234	437	720	653	675	564	3,458	
(%)	40.1	44.3	48.3	48.2	48.8	39.9	32.9	26.1	37.9	

Men										
Total deaths	98	155	319	584	957	951	1,221	1,187	5,472	
Cancer deaths	33	61	145	270	481	396	405	354	2,145	100.0
(%)	33.7	39.4	45.5	46.2	50.3	41.6	33.2	29.8	39.2	

Cancer site:										
Lung	8	7	26	48	124	100	91	83	487	22.7
Stomach	9	15	27	62	92	87	88	71	451	21.0
Liver	2	8	35	46	76	44	48	30	289	13.5
Large intestine	5	11	16	31	35	31	34	35	198	9.2
Pancreas	2	4	9	11	24	23	27	29	129	6.0
Gallbladder/bile duct	0	2	4	17	23	30	16	16	108	5.0
Prostate	0	0	0	2	10	15	24	30	81	3.8
Esophagus	3	2	6	13	20	13	11	7	75	3.5
Others	4	12	22	40	77	53	66	53	327	15.2

Women										
Total deaths	59	98	165	322	517	686	830	976	3,653	
Cancer deaths	30	51	89	167	239	257	270	210	1,313	100.0
(%)	50.8	52.0	53.9	51.9	46.2	37.5	32.5	21.5	35.9	

Cancer site:										
Stomach	4	9	7	33	40	46	43	38	220	16.8
Lung	6	3	12	18	30	33	36	20	158	12.0
Large intestine	2	6	10	18	26	30	31	31	154	11.7
Pancreas	0	2	7	12	28	22	28	20	119	9.1
Gallbladder/bile duct	1	7	8	14	16	19	30	21	116	8.8
Liver	1	4	3	16	21	35	17	17	114	8.7
Breast	6	10	11	12	17	9	9	4	78	5.9
Ovary	3	4	8	5	14	4	6	4	48	3.7
Others	7	6	23	39	47	59	70	55	306	23.3

## FRAMEWORKS INTENDED FOR STATISTICAL ANALYSIS

The JACC Study Group has decided to organize several study subgroups to examine site-specific risk modification by lifestyle variable based on the follow-up data as of the end of 1997, and has started to analyze the data for such major sites as lung, stomach, pancreas and gallbladder/bile duct, of which epidemiological data analyses were almost at final stage at moment. The central office of Monbusho ECC was agreed by all the investigators to be responsible for analyzing risk modification by lifestyle variables for total deaths and total cancer deaths. An analysis will be started for such sites as cancers of the large intestine and liver in the fiscal year of 2002 under the financial supports from Monbusho.

In our JACC Study we have stored sera of about 39,000 cohort members at -80°C. The JACC Study Group has recently decided and started to measure, at one reliable laboratory (SRL: Hachioji, Japan), superoxidedismutase (SOD) activity, insulin-like growth factor-I (IGF-I), insulin-like growth factor-II (IGF-II), insulin-like growth factor-binding protein 3 (IGF-BP3), soluble Fas (sFas), and transforming growth factor-β1 (TGF-β1) among all the deceased and incident cancer sufferers as well as their triple healthy controls (about 15,000 samples in total). This measurement was expected to be finished at the end of the fiscal year of 2000. For site-specific analysis, we have decided to measure other biological makers for all the deceased and incident cancer sufferers by site and their double or triple healthy controls. The measured values will be included in an analysis by a site-specific nested case-control study.

Biological markers to be measured for site-specific nested case-control studies are not finally, but tentatively decided, and are going to be measured. Biological markers probably to be measured are folic acid, total cholesterol, tumor necrozing factor-*α* (TNF-*α*), carotenoids, 8-hydroxy-2′-deoxy-guanosine, Mn-SOD and others for a nested case-control study on lung cancer. For a nested case-control study on stomach cancer, *Helicobactor Pylori* antibody, pepsinogen I and II, total cholesterol, carotenoids, zinc and others are to be measured. Those to be measured are fructosamine, albumin, insulin, total cholesterol, total fatty acid fractions, and such metals as selenium, zinc and cooper for a nested case-control study on pancreatic cancer, and total cholesterol, HDL-cholesterol, interleukin-6, pepsinogen I and II, *Helicobactor Pylori* antibody, total fatty acid fractions and such metals as cadmium, chromium, and selenium for that on cancer of the gallbladder and bile duct. For a nested case-control study on cancer of the large intestine, which will be started in 2002, insulin, fructosamine, albumin, HDL-cholesterol, folic acid, carotenoids, and total fatty acid and bile acid fractions are tentatively decided as biological markers to be measured. Possible studies that will include DNA are not yet planned because of ethical issues to be resolved.

The site-specific study design on cancers of the lung, stomach and liver, including a nested case-control study design, which would use both epidemiological and biological materials (serum only), singularly or collectively, was approved by the ethical board at the university to which each principal investigator is affiliated.

## MEMBERS OF JACC STUDY GROUP

The present investigators involved, with the co-authorship of this paper, in the JACC Study and their affiliations are as follows: Dr. Yoshiyuki Ohno (the present chairman of the study group), Nagoya University Graduate School of Medicine; Dr. Mitsuru Mori, Sapporo Medical University School of Medicine; Dr. Yutaka Motohashi, Akita University School of Medicine; Dr. Shigeru Hisamichi, Tohoku University Graduate School of Medicine; Dr. Yosikazu Nakamura, Jichi Medical School; Dr. Takashi Shimamoto, Institute of Community Medicine, University of Tsukuba; Dr. Haruo Mikami, Chiba Cancer Center; Dr. Shuji Hashimoto, School of Health Sciences and Nursing, University of Tokyo; Dr. Yutaka Inaba, Juntendo University School of Medicine; Dr. Heizo Tanaka, Medical Research Institute, Tokyo Medical and Dental University; Dr. Yoshiharu Hoshiyama, Showa University School of Medicine; Dr. Hiroshi Suzuki, Niigata University School of Medicine; Dr. Hiroyuki Shimizu, Gifu University School of Medicine; Dr. Hideaki Toyoshima, Nagoya University Graduate School of Medicine; Dr. Akiko Tamakoshi, Nagoya University Graduate School of Medicine; Dr. Shinkan Tokudome, Nagoya City University Medical School; Dr. Yoshinori Ito, Fujita Health University School of Health Sciences; Dr. Akio Koizumi, Graduate School of Medicine and Faculty of Medicine Kyoto University; Dr. Takashi Kawamura, Kyoto University Center for Student Health; Dr. Yoshiyuki Watanabe, Kyoto Prefectural University of Medicine, Research Institute for Neurological Diseases & Geriatrics; Dr. Masahiro Nakao, Kyoto Prefectural University of Medicine; Dr. Takaichiro Suzuki, Research Institute Osaka Medical Center for Cancer and Cardiovascular Diseases; Dr. Tsutomu Hashimoto, Wakayama Medical College; Dr. Takayuki Nose, Tottori University Faculty of Medicine; Dr. Norihiko Hayakawa, Research Institute for Radiation Biology and Medicine, Hiroshima University; Dr. Takesumi Yoshimura, Institute of Industrial Ecological Sciences, University of Occupational and Environmental Health, Japan; Dr. Katsuhiro Fukuda, Kurume University School of Medicine; Dr. Naoyuki Okamoto, Kanagawa Cancer Center; Dr. Teruo Ishibashi, Asama General Hospital; Dr. Hideo Shio, Shiga Medical Center; Dr. Tomoyuki Kitagawa, Cancer Institute, Tokyo; Dr. Toshio Kuroki, Institute of Molecular Oncology, Showa University; and Dr. Kazuo Tajima, Aichi Cancer Center Research Institute.
